# A flexible speller based on time-space frequency conversion SSVEP stimulation paradigm under dry electrode

**DOI:** 10.3389/fncom.2023.1101726

**Published:** 2023-02-01

**Authors:** Ze Zhang, Dandan Li, Yao Zhao, Zhihao Fan, Jie Xiang, Xuedong Wang, Xiaohong Cui

**Affiliations:** College of Information and Computer, Taiyuan University of Technology, Taiyuan, China

**Keywords:** brain-computer interface (BCI), time-space frequency conversion (TSFC) SSVEP, brain-controlled switch, electrooculography (EOG), dry electrode

## Abstract

**Introduction:**

Speller is the best way to express the performance of the brain-computer interface (BCI) paradigm. Due to its advantages of short analysis time and high accuracy, the SSVEP paradigm has been widely used in the BCI speller system based on the wet electrode. It is widely known that the wet electrode operation is cumbersome and that the subjects have a poor experience. In addition, in the asynchronous SSVEP system based on threshold analysis, the system flickers continuously from the beginning to the end of the experiment, which leads to visual fatigue. The dry electrode has a simple operation and provides a comfortable experience for subjects. The EOG signal can avoid the stimulation of SSVEP for a long time, thus reducing fatigue.

**Methods:**

This study first designed the brain-controlled switch based on continuous blinking EOG signal and SSVEP signal to improve the flexibility of the BCI speller. Second, in order to increase the number of speller instructions, we designed the time-space frequency conversion (TSFC) SSVEP stimulus paradigm by constantly changing the time and space frequency of SSVEP sub-stimulus blocks, and designed a speller in a dry electrode environment.

**Results:**

Seven subjects participated and completed the experiments. The results showed that the accuracy of the brain-controlled switch designed in this study was up to 94.64%, and all the subjects could use the speller flexibly. The designed 60-character speller based on the TSFC-SSVEP stimulus paradigm has an accuracy rate of 90.18% and an information transmission rate (ITR) of 117.05 bits/min. All subjects can output the specified characters in a short time.

**Discussion:**

This study designed and implemented a multi-instruction SSVEP speller based on dry electrode. Through the combination of EOG and SSVEP signals, the speller can be flexibly controlled. The frequency of SSVEP stimulation sub-block is recoded in time and space by TSFC-SSVEP stimulation paradigm, which greatly improves the number of output instructions of BCI system in dry electrode environment. This work only uses FBCCA algorithm to test the stimulus paradigm, which requires a long stimulus time. In the future, we will use trained algorithms to study stimulus paradigm to improve its overall performance.

## 1. Introduction

Brain-computer interface (BCI) is currently a research hotspot in the multidisciplinary cross-field (Abiri et al., 2019; Mcfarland, 2020; Pan et al., 2020), which aims to establish a direct communication channel between the brain and external devices without relying on peripheral nerve and muscle tissue (Zhou et al., 2014; Edelman et al., 2019; Li et al., 2019; Jin et al., 2020; Lu et al., 2020; Ge et al., 2021). At present, the BCI system is widely used in medical rehabilitation, mechanical control, speller, etc. BCI speller is the best way to express the performance of the BCI paradigm (Kapgate et al., 2020; Li et al., 2021a). Many researchers have attempted to implement multi-instruction BCI spellers to improve the information transmission rate (ITR) of the BCI system (Rezeika et al., 2018; Xu et al., 2020; Li et al., 2021a; Kundu and Ari, 2022). Therefore, it is of great significance for the development of BCI to study the speller that outputs multiple instructions in a short time. However, considering the actual use of patients with motion disorders, the system design should improve the convenience of patients while ensuring the accuracy of instructions.

The BCI speller is a typical visual application of the BCI and was among the earliest implementations of the concept. Steady-state visual-evoked potential (SSVEP) (Zhang and Chen, 2022) is often used to design spellers because of its stable evoked characteristics and high signal-to-noise ratio. Nakanishi et al. proposed an SSVEP speller based on an FPHC paradigm to design the speller (Nakanishi et al., 2014). Xu et al. designed a 108 characters speller based on the hybrid paradigm of P300 and SSVEP in 2020 (Xu et al., 2020). In 2021, Ge et al. designed a 48-instruction speller based on the dual frequency SSVEP-biased coding paradigm (Ge et al., 2021). Although there are many speller studies on SSVEP, those speller systems can only be completed in the laboratory environment using wet electrodes, which are cumbersome and inflexible, and the subject experience is poor.

The dry electrode (Li et al., 2021b) based on high comfort and good portability has become the research focus of various BCI systems. In 2016, Chi Chun Lo et al. designed a new non-contact 12-instruction SSVEP control system, allowing disabled patients to activate the nurse emergency call system and adjust other equipment (Lo et al., 2016). In 2018, Xiao et al. used claw-shaped flexible dry electrodes to design and implement a speller with 12 targets (Xing et al., 2018), with an average accuracy of 93.2%, which is convenient for the subjects to complete character output in any environment. At present, although there are studies based on dry electrode SSVEP, the research is not mature enough and there are few output instructions.

In recent years, many researchers have begun to design and implement BCI systems based on asynchronous SSVEP (Pfurtscheller et al., 2010; Diez et al., 2011) in order to improve the flexibility of the system. Some studies use the threshold value of the stimulus flicker as the standard (Pan et al., 2013; Zhou et al., 2020). When the stimulus flickers continuously since the beginning of the experiment (including the control and idle state), the long-time flicker will make the subjects feel strong fatigue. Because the EOG signal is easy to be detected, it is widely concerned. Some studies combine EOG with MI to reflect the intention of the subjects and send commands to external devices. In 2017, He et al. proposed a hybrid BCI based on MI and EOG signals to operate web browsers (He et al., 2017). In 2019, Huang et al. used EOG for button selection and MI for direction control to integrate the control of the wheelchair robot arm system (Huang et al., 2019). Although EOG performs well in the MI paradigm, there are few studies that combine EOG with SSVEP and even fewer studies that use EOG to improve the flexibility of the SSVEP system.

This study designed and implemented a speller that can output multiple instructions flexibly. We designed a brain-controlled switch based on EOG and SSVEP. By using the brain-controlled switch to wake up and turn off the flashing stimulus, no external stimulus is needed in the idle state, which reduces the visual fatigue caused by flashing and is more consistent with the perception of the idle state. In addition, this study designed a time-space frequency conversion (TSFC) SSVEP stimulation paradigm based on the Neuracle 24-channel dry electrode and increased the number of output instructions within the limited frequency range by constantly changing the frequency of SSVEP sub-stimulation block space in two stages. First, we divided the stimulus interface into eight stimulus regions, which can accurately identify the target stimulus region in a short time. Second, the spatial local flicker designed to reduce the influence of irrelevant stimulus blocks as much as possible and effectively improve the accuracy of stimulus block recognition. The speller system based on our proposed TSFC-SSVEP paradigm can output 60 characters accurately. The main contribution of this study is to design a flexible speller combined with a brain-controlled switch, which provides flexibility and comfort. This study proposed the TSFC-SSVEP stimulation paradigm that can make speller output multiple instructions under the dry electrode. The study provides a new idea for the speller based on the brain-controlled system.

## 2. Methods

### 2.1. Participants

In this study, seven healthy subjects (aged 25.14 ± 0.98 years) volunteered to participate in offline and online experiments, and all subjects had normal vision or corrected vision. The experiment was conducted in a quiet laboratory. In addition, before each experiment, the experimenter informed the subjects of SSVEP-related theoretical knowledge and precautions for looking at the screen. All subjects had informed consent to the experimental study, and the study was approved by the Ethics Committee of the Taiyuan University of Technology.

### 2.2. Data acquisition

The dry electrode experiment part of this study uses the Neuracle 24-channel dry electrode, which is designed as a claw-like structure. The electrode tip is coated with a silver/silver chloride mixture of conductive ink to improve the electrochemical performance. The structure and material characteristics make it lightweight and wear-resistant. The wet electrode experiment part uses the Brain Products GmbH 32 moisture-conducting electrode made in Germany. The dry electrode can pass through the hair and contact the scalp well, and the correlation with the wet electrode signal is >90%.

The EEG signal sampling rate is 300 Hz. Six electrodes in the parietal occipital region (P3, P4, T5, T6, O1, and O2) and four electrodes in the frontal lobe (Fp1, Fp2, F7, and F8) were recorded. The reference electrode is located at the top of the head (PFz), and the grounding electrodes are the A1 and A2 of the earlobe. The impedance of all electrodes is < 50 *kΩ*.

### 2.3. Paradigms design

**Figure 1:**
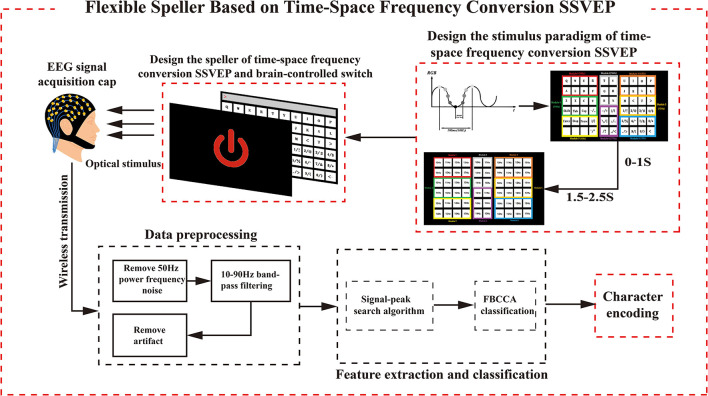
Experimental technology roadmap.

As shown in Figure 1, this paradigm is divided into two stages. First, the brain-controlled switch is designed to control the system flexibly. Second, the design is based on the TSFC-SSVEP stimulation paradigm to increase the number of BCI instruction codes in the dry electrode environment. Finally, the two are integrated to design a flexible and comfortable speller for the subjects.

#### 2.3.1. Design of brain-controlled switch

The brain-controlled switch is composed of EOG and SSVEP. The EOG in the brain-controlled switch is used to wake up stimulus because the EOG is easy to detect. EOG is the depolarization and hyperpolarization between the retina and cornea caused by different eye movements, which forms a potential difference between the retina and cornea, and its amplitude is greater than that of EEG and background physiological signals (Crea et al., 2018; Huang et al., 2019; Zhu et al., 2020). Therefore, EOG can be easily and accurately detected by using several electrodes around the eyes. When the subject blinks continuously, we consider that the subject intends to wake up the stimulus. The SSVEP in the brain-controlled switch is used to turn off system stimulation. When the subjects are looking at the “Stop” SSVEP stimulus block in the stimulation keyboard, the stimulation can be turned off.

**Figure 2:**
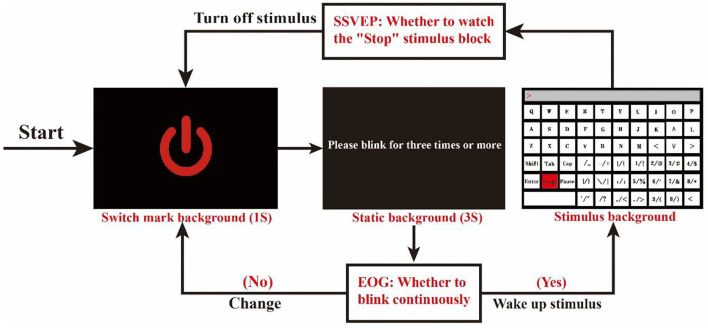
Schematic diagram of the brain-controlled switch.

As shown in Figure 2, the system will first enter the switch mark background, which lasts for 1 s. This background is a sign to prompt the subject to open the stimulus. Then, the system will switch to a static background, with a duration of 3 s. Within 3 s, the subject can complete the operation of whether to wake up the stimulus according to their own intentions. If the subject blinks three times or more, the system switches to the stimulation keyboard interface, and vice versa. The three blinks here are the thresholds we set, mainly because the normal blinking of the subjects is excluded. Under normal conditions, the subjects also have a high probability of having 1–3 physiological blinks in a 3-s period (Zhu et al., 2020). Finally, after the subject wakes up the stimulation keyboard, he/she can output the required characters according to his/her intention. When all characters are output, the subject looks at the “Stop” SSVEP stimulus block on the stimulation keyboard to close the stimulation keyboard, and the system will switch to the background of the switch mark.

#### 2.3.2. Design of TSFC-SSVEP stimulation paradigm

**Figure 3:**
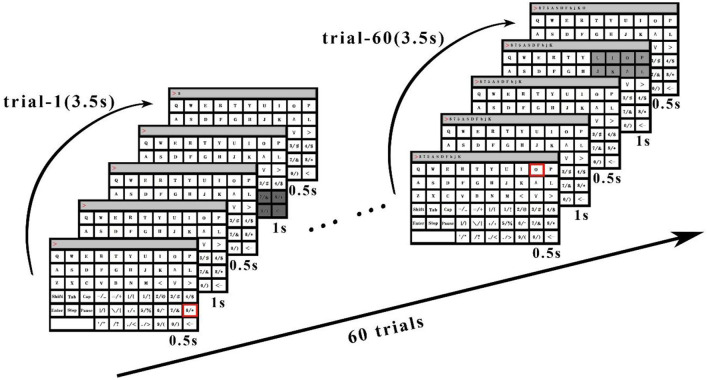
TSFC-SSVEP stimulation paradigm timeline.

**Figure 4:**
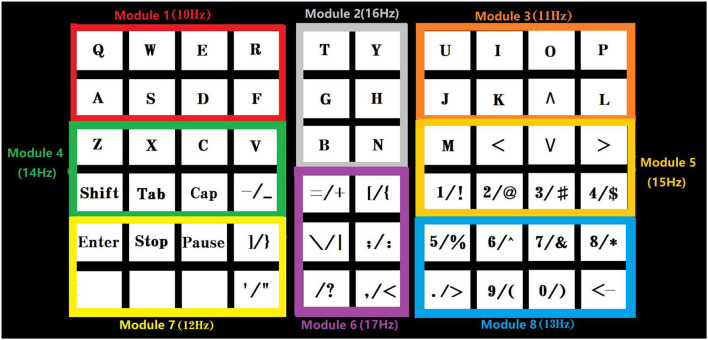
Schematic diagram of the first stage of TSFC-SSVEP stimulation paradigm.

**Figure 5:**
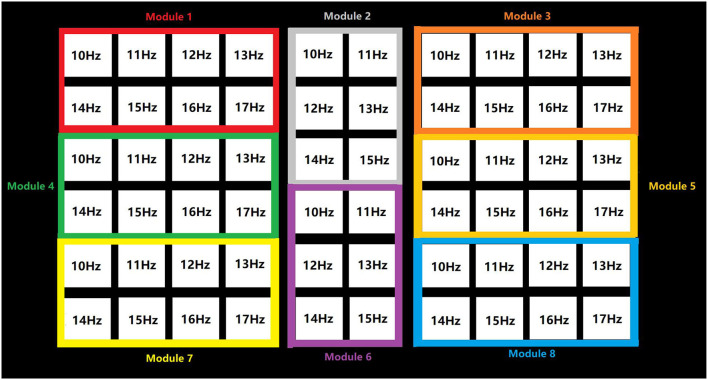
Schematic diagram of the second stage of the TSFC-SSVEP stimulation paradigm.

As shown in Figure 3, the stimulation time of the TSFC-SSVEP paradigm in this study is 2 s. In the offline experiment, the character cue time is 0.5 s (the red square is marked with prompted characters), the stimulus shift time is 0.5 s, and the character feedback time is 0.5 s, with a total of 3.5 s. In the online experiment, the time of stimulus shift was 0.5 s and the time of character feedback was 0.5 s, with a total of 3 s. The specific stimulation is divided into two stages. The duration of the first stage stimulation is 1 s. As shown in Figure 4, the stimulation interface is divided into eight large modules corresponding to the frequency ranging from 10 to 17 Hz of the sine wave. The reasons for the selection of the 10–17 Hz range are specified in the results section. The spatial range of the subjects' gaze can be determined by analyzing the frequency of the modules within 1 s. The 1–1.5 s is the stimulus conversion process, which feeds back the spatial position analyzed in the first stage to the interface and transfers to the second stage of the stimulus interface. As shown in Figure 5, the 1.5–2.5 s is the second-stage stimulus. The stimulus is frequency coded again in each module of the first stage. The stimulus process is only carried out within the scope of the first-stage analysis, and other modules remain unchanged. As shown in Figure 3, the gray area is that selected by the subject in the first stage. Among them, compared with medium frequency (15–30 Hz) and high frequency (>30 Hz) stimuli, medium and low frequency (about 15 Hz) stimuli can induce stronger SSVEP responses. Therefore, in this paradigm, the stimulation frequency range of the stimulation flicker block is selected as the medium- and low-frequency band.

The frequency conversion combination of SSVEP stimuli in time and space is carried out according to the above two stages, which can encode different instructions, greatly improving the performance of the SSVEP paradigm. Moreover, the signal-to-noise ratio of feature signals is high, and the requirement for display is low, which is convenient for target recognition.

### 2.4. Signal processing

In this article, the features extracted by the speller are divided into two categories, one is EOG features, and the other is the SSVEP feature of EEG. Among them, the EOG feature extraction is divided into three steps. First, we use 0.5–10 Hz band-pass filtering for preprocessing. Then, the data of the four channels (Fp1, Fp2, F7, and F8) are averaged. Finally, the peak value above the 0.07 mV threshold is extracted by using the signal-peak search algorithm. In the signal-peak search algorithm, a peak or local maximum is defined as any sample whose two direct neighbors have a smaller amplitude. When the number of peaks is >3, it can be judged as conscious blinking, otherwise, it is normal. In addition, the feature extraction of SSVEP in EEG is divided into two steps. The first is preprocessing, i.e., 10–90 Hz band-pass filtering, 50 Hz notch processing, and ICA artifact removal. The second is to classify the filter bank canonical correlation analysis (FBCCA) (Chen et al., 2015a). First, the FBCCA is used to analyze the stimulus block region that the subject is currently looking at, then the FBCCA is used to analyze the specific stimulus block of the subject's gaze region, and finally, the feature fusion analysis is performed to obtain the final character output result.

Filter bank analysis uses multiple filters with different passbands for sub-band decomposition. A zero-phase Chebyshev *I* infinite pulse filter is used to extract the sub-band component*X*_*S*_*B*__*n*__(*n* = 1, 2, …, *N*) from the original EEG signal X. After the filter bank analysis, the standard Canonical Correlation Analysis (CCA) (Lin et al., 2007; Bin et al., 2009; Chen et al., 2014) process is applied to each sub-band component, respectively, and the correlation values between the sub-band component and the predefined reference signals corresponding to all stimulus frequencies *Y*_*fk*_ are obtained. For the kth reference signal, a correlation vector ρ_*k*_ composed of N correlation values is defined as follows:


(1) $$\begin{array}{l} \left[\begin{array}{c} \rho_{k}^{1}\\ \rho_{k}^{2}\\ \vdots \\ \rho_{k}^{N} \end{array}\right]=\left[\begin{array}{c} \rho \left[X_{SB_{1}}^{T}W_{X}\left(X_{SB_{1}}Y_{fk}\right),Y^{T}W_{Y}\left(X_{SB_{1}}Y_{fk}\right)\right]\\ \rho \left[X_{SB_{2}}^{T}W_{X}\left(X_{SB_{2}}Y_{fk}\right),Y^{T}W_{Y}\left(X_{SB_{2}}Y_{fk}\right)\right]\\ \vdots \\ \rho \left[X_{SB_{N}}^{T}W_{X}\left(X_{SB_{N}}Y_{fk}\right),Y^{T}W_{Y}\left(X_{SB_{N}}Y_{fk}\right)\right] \end{array}\right] \end{array}$$

In formula (1), ρ(*x, y*) represents the correlation coefficient between *x* and *y*. The weighted sum of squares of the correlation values of all corresponding sub-band components is calculated as follows:


(2) $$\begin{array}{l} {\tilde{\rho }}_{k}=\overset{N}{\underset{n=1}{\sum }}\omega \left(n\right){\left(\rho_{k}^{n}\right)}^{2} \end{array}$$

In formula (2), *n* represents the index of the sub-band; ω(*n*) is the weight of the sub-band component, and the calculation formula is given as follows:


(3) $$\begin{array}{l} \omega \left(n\right)=n^{-a}+b,n\in \left[1,N\right] \end{array}$$

In formula (3), *a* and *b* are constants that maximize the classification performance, which can be determined by using the grid search method in offline analysis. For all stimulus frequencies $\left({\tilde{\rho }}_{1},{\tilde{\rho }}_{2},\ldots ,{\tilde{\rho }}_{N}\right)$, ${\tilde{\rho }}_{k}$ is used to determine the frequency of SSVEP. When ${\tilde{\rho }}_{k}$ is the maximum, the frequency of the reference signal is the frequency of SSVEP.

Compared with standard CCA, the filter group analysis in FBCCA can decompose SSVEP into multiple sub-band components, so as to extract the discriminant information in the harmonic components of SSVEP. Therefore, FBCCA provides richer and more robust harmonic information for SSVEP target recognition and has a better recognition effect.

Based on previous research (Chen et al., 2015a), this article selects the best M3 sub-band division FBCCA method. The specific process is as follows:

Step 1: Divide the effective frequency band (10–90 Hz) of the signal into 10 segments (10–90, 18–90, 26–90, 34–90, 42–90, 50–90, 58–90, 66–90, 74–90, and 82–90 Hz);

Step 2: Pass the collected EEG signals through the above 10 band-pass filters, respectively;

Step 3: Substitute the 10 groups of EEG signals into the 10 standard CCAs, and then calculate the maximum correlation coefficient sum through the weight adjustment formula. The frequency corresponding to the maximum correlation coefficient sum is recorded as the prediction frequency.

## 3. Results

### 3.1. Brain-controlled switch experiment classification results

Brain-controlled switch experiment is divided into the offline experiment and the online experiment. In the experiment on EOG signal wake-up system stimulation, the online experiment and offline experiment were divided into four rounds, each round had 10 consecutive blinks and 10 normal states. Through the analysis and processing of the data obtained from the offline experiment, we set the corresponding signal peak threshold to 0.07 mV and the peak frequency threshold to 3 times. As shown in Table 1, seven subjects conducted offline online experiments. Among them, the accuracy rate of three subjects in the offline experiment was as high as 100.00%, and the accuracy rate of only one subject was 85.00%, with an average accuracy rate of 95.89%. Under the threshold determined by the offline experiment, the two subjects performed well in the online experiment, with an accuracy rate of 100.00% and an average accuracy rate of 94.64%, which fully conforms to our expected experimental hypothesis. In the experiment for the SSVEP signal to turn off the system stimulus, we selected the accuracy of seven subjects in 60 stimulus blocks of the entire stimulate keyboard (refer to the “TSFC-SSVEP Speller Classification Results” section for the specific experiment process). The seven subjects can complete the switching operation of the system stimulus according to their own intentions, which also conforms to our expected experimental hypothesis. The main purpose of the brain-controlled switch is to make the subject more autonomous in the process of SSVEP stimulation. In addition, the brain-controlled switch can avoid the visual fatigue of the subject caused by the long-time flicker after the SSVEP stimulation is turned on.

**Table 1:** Classification accuracy of the brain-controlled switch experiment.

**Subject**	**EOG (Wake up stimulus) Accuracy**		**SSVEP (Turn off stimulus) Accuracy**	

	**Offline (%)**	**Online (%)**	**Offline (%)**	**Online (%)**
S1	90.00	87.50	96.65	97.50
S2	100.00	98.75	90.00	90.00
S3	100.00	95.00	90.40	92.50
S4	100.00	100.00	83.75	90.00
S5	85.00	88.75	91.65	90.00
S6	98.75	92.50	90.85	95.00
S7	97.50	100.00	87.93	90.00
Mean ± SD	95.89 ± 5.53	94.64 ± 4.85	90.18 ± 3.60	92.14 ± 2.81

### 3.2. Determination of SSVEP characteristic frequency and time window

**Figure 6:**
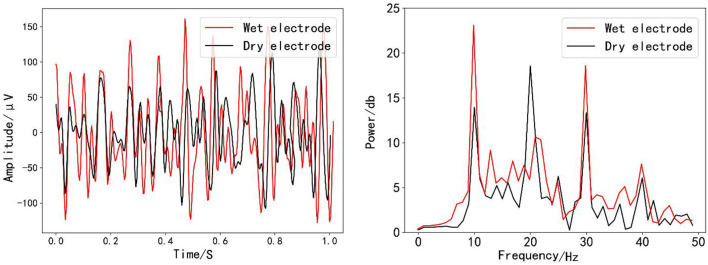
Dry and wet electrodes: **(A)** time domain comparison diagram and **(B)** frequency domain comparison diagram.

In this experiment, dry and wet electrodes were compared in the time domain and frequency domain, respectively. As shown in Figure 6A, in the time domain, after the dry electrode and wet electrode occipital leads are superposed and averaged, respectively, the signals are processed by 10–50 Hz band-pass filtering, and the two signals can be basically matched. As shown in Figure 6B, on the power spectrum, after the dry electrode and wet electrode pillow leads are superposed and averaged, respectively, the signals are Fourier transformed. The characteristics of both signals at the fundamental frequency signal and the harmonic signal are obvious. In conclusion, the dry electrode conditions used in this experiment basically meet the experimental requirements, and subsequent experiments are carried out based on dry electrodes.

Table 2 shows the recognition accuracy of seven subjects using dry electrodes for eight categories of SSVEP (8–15 Hz) at different time windows. Among them, the accuracy rate of only two subjects in 0.4 s was more than 90.00%, while in 1 s, all subjects reached 90.00%, and the average accuracy rate was 95.61%. The results in Table 2 show that the recognition accuracy increases with the increase of the time window, that is, the larger the time window is. It can achieve good results for the eight classification results in ~1 s, which provides a reliable experimental basis for the TSFC-SSVEP experiment.

**Table 2:** Recognition accuracy of dry electrodes in different time windows experiment.

**Subject**	**Accuracy (%) on 0.4s**	**Accuracy (%) on 0.6s**	**Accuracy (%) on 0.8s**	**Accuracy (%) on 1s**
S1	91.70	98.30	96.70	100.00
S2	73.30	75.00	85.00	90.00
S3	81.70	91.70	96.70	100.00
S4	96.70	98.30	100.00	100.00
S5	56.70	88.30	96.70	93.30
S6	70.80	84.15	92.50	93.30
S7	72.08	87.08	90.08	92.70
Mean ± SD	77.57 ± 12.61	88.98 ± 7.60	93.95 ± 4.73	95.61 ± 3.94

In the process of frequency selection, we designed the online experiment of TSFC-SSVEP in the 8–15 Hz frequency band, but the experimental results were not ideal. According to the feedback results of the subjects after the experiment, we made further analysis. The pixel size of 60 stimulus blocks is much smaller than that of 8 stimulus blocks, which results in reducing the induced SSVEP characteristics. In addition, the subjects found that higher frequency (>10 Hz) stimuli could attract the subjects' attention. This led to the involuntary transfer of subjects' attention from a low-frequency stimulus to a high-frequency stimulus when they looked at the stimulus block in the second stage, which affected the experimental results. Hence, we designed online experiments to screen the frequencies from 10 to 20 Hz, respectively, and 7 subjects focused on 60 stimulus blocks, and finally, 10–17 Hz is determined as a better frequency range. Table 3 shows the classification results of the TSFC-SSVEP online experiment of 7 subjects in 8–15 and 10–17 Hz bands, respectively. In this experiment, in order to avoid the influence of the time window on the experimental results, we used a 2-s stimulation time window in both stimulation stages. The results showed that the performance of the seven subjects under the stimulation paradigm of TSFC-SSVEP realized in the higher frequency band was higher than that in the lower frequency band. Therefore, we determined that the frequency range of TSFC-SSVEP was 10-17 Hz.

**Table 3:** Online results of TSFC-SSVEP stimulation paradigm at two different frequencies.

**Subject**	**Accuracy (%) of 8–15Hz**	**Accuracy (%) of 10–17Hz**
S1	91.67	98.33
S2	83.33	91.67
S3	86.67	93.33
S4	76.67	85.00
S5	88.33	96.67
S6	86.67	95.00
S7	80.00	90.00
Mean ± SD	84.76 ± 4.75	92.86 ± 4.15

### 3.3. TSFC-SSVEP speller classification results

The data from 7 healthy subjects were collected in this experiment. In the offline experiment, each subject selects 60 characters in each round, that is, 60 trials and the duration of a trial is 3.5 s. During the online experiment, 10 random characters specified by the experimenter shall prevail, that is, 10 trials, with a trial duration of 3 s. Each subject conducted 4 rounds of experiments offline and online, namely, 4 blocks, and the final result was the average of offline and online results. Table 4 shows the accuracy and ITR of 7 subjects using the FBCCA classification algorithm under the TSFC-SSVEP stimulation paradigm. It can be seen from Table 4 that the accuracy of most subjects in the offline experiment is more than 90.00%, the average accuracy of the offline experiment is 90.18%, while the average accuracy of the online experiment is 92.14%, with a difference of 2.00%. We analyzed the reasons and combined them with the feedback information of the subjects, mainly because the number of 60 stimulus targets in a round was large, and the long-term stimulus flickering led to visual fatigue of the subjects, which ultimately affected the experimental results.

**Table 4:** Classification results based on TSFC-SSVEP in FBCCA algorithm.

**Subject**	**Accuracy (%)**		**ITR (bits/min)**	

	**Offline**	**Online**	**Offline**	**Online**
S1	96.65	97.50	131.96	134.19
S2	90.00	90.00	116.39	116.39
S3	90.40	92.50	117.26	121.95
S4	83.75	90.00	103.46	116.39
S5	91.65	90.00	120.03	116.39
S6	90.85	95.00	118.25	127.83
S7	87.93	90.00	111.97	116.39
Mean ± SD	90.18 ± 3.60	92.14 ± 2.81	117.05 ± 7.96	121.36 ± 6.61

### 3.4. Different paradigms comparison results

In this experiment, the TSFC-SSVEP paradigm and the current popular Joint Frequency Phase Modulation (JFPM)-SSVEP (Chen et al., 2015b) paradigm were analyzed and compared offline under the same time window for the same subjects. Table 5 shows the offline accuracy of character recognition of 7 subjects using the FBCCA algorithm under 2 different paradigms. JFPM-SSVEP is not as effective as it is in a wet electrode environment. The accuracy rate of the 2 subjects was lower than 80.00%, and there was a large difference between different subjects. The TSFC-SSVEP proposed in this article has a stable effect in the dry electrode environment. Only two subjects have a result lower than 90.00%, and there is less difference between different subjects. The results showed that compared with the JFPM-SSVEP paradigm, the TSFC-SSVEP stimulus paradigm had more advantages in the case of more instructions in the dry electrode environment, and its stability, accuracy, and ITR were higher than those of JFPM-SSVEP.

**Table 5:** Classification results of FBCCA algorithm under different stimulus paradigms.

**Subject**	**JFPM Accuracy (%)**	**TSFC Accuracy (%)**	**JFPM ITR (bits/min)**	**TSFC ITR (bits/min)**
S1	95.00	96.65	114.51	131.96
S2	92.50	90.00	108.99	116.39
S3	90.00	90.40	103.78	117.26
S4	67.50	83.75	66.66	103.46
S5	77.50	91.65	80.72	120.03
S6	82.50	90.85	89.47	118.25
S7	87.50	87.93	98.82	111.97
Mean ± SD	84.64 ± 8.91	90.18 ± 3.60	94.71 ± 15.60	117.05 ± 7.96

## 4. Discussion

This study designed and implemented a multi-instruction SSVEP speller based on dry electrodes. At the same time, through the combination of EOG and SSVEP signals in BCI, the speller can be flexibly controlled. The TSFC-SSVEP stimulation paradigm proposed in this study recodes the frequency of SSVEP stimulus sub-blocks in time and space, reduces the influence of surrounding stimulus blocks by using local flicker, performs well under dry electrode testing, and greatly improves the output command of the BCI system. Compared with synchronous BCI, the system uses a brain-controlled switch based on EOG and SSVEP to wake up or turn off stimulus flicker to complete character output, which makes the system more flexible and convenient.

As for asynchronous BCI systems, the previous research on asynchronous SSVEP-based BCI mainly uses traditional threshold methods to distinguish between the control state and the idle state (Pfurtscheller et al., 2010). In these studies, even if the subject is in an idle state, the stimulus in the stimulus interface will flicker continuously from the beginning of the experiment, which is easy to cause visual fatigue in the subject. Several researchers designed novel methods to improve the performance of the asynchronous SSVEP-based BCI. Pfurtscheller et al. used an MI-based brain switch to achieve the self-paced operation of an SSVEP-based orthosis control system. Tomita et al. proposed a bimodal BCI using simultaneously NIRS and EEG signals to estimate whether the subject is in idle or active mode (Tomita et al., 2014). In this study, we used EOG in combination with SSVEP to awaken or turn off systemic stimuli. The subject wakes up or closes the asynchronous operation based on the TSFC-SVEP speller according to their intention. Compared with asynchronous systems using threshold criteria, BCI systems based on EOG and SSVEP do not need to continuous flashing in idle state. In the idle state, no stimulation can alleviate the visual fatigue of the subjects. In addition, compared to MI-based switches, brain-controlled switches based on EOG and SSVEP have the advantage of short response time, which can accurately distinguish between the control state and idle state in a short time, and effectively improve system performance. But the brain-controlled switch based on EOG and SSVEP also has its limitations. For different subjects, the main challenge is that the system needs to collect a round of subjects' blink data in advance because different subjects have different blink ranges. Therefore, future work needs to design a more robust algorithm to classify the blinks of different subjects.

For the BCI classification based on TSFC-SSVEP, we have only tested the FBCCA algorithm. However, relying on FBCCA no-training algorithm analysis requires a time window of at least 1 s, which limits the performance of the stimulus paradigm. According to recent research, TRCA (Jin et al., 2021; Bian and Wu, 2022) and FBDNN (Bassi and Attux, 2022) can shorten the time window of stimulus data to 0.5 s, which will better improve the performance of the stimulus paradigm. We will also study the corresponding algorithm in the future.

## 5. Conclusion

In this article, a speller based on the brain-controlled switch and TSFC-SVEP stimulation paradigm is designed using a dry electrode. The brain-controlled switch controls the wake-up or turn off of the system stimulus by capturing the blink state of the subject and whether the subject looks at the “Stop” SSVEP stimulus block in the stimulus keyboard, which greatly improves the flexibility of the system. At the same time, the TSFC-SSVEP experimental stimulation paradigm proposed in this article greatly increases the number of instructions in the stimulation paradigm by combining SSVEP sub-stimulation modules in time and space and uses the idea of local stimulus block flashing to avoid irrelevant influence, which improves the recognition accuracy in a short time. The results show that the EOG recognition accuracy of the brain-controlled switch designed in this study is as high as 94.64%, and all subjects can flexibly use the speller. The speller based on TSFC-SSVEP can output 60 characters with an accuracy rate of 90.18%, It opens the way for the portable and comfortable dry electrode BCI system in the future.

## Data availability statement

The raw data supporting the conclusions of this article will be made available by the authors, without undue reservation.

## Ethics statement

The studies involving human participants were reviewed and approved by Ethics Committee of Taiyuan University of Technology. The patients/participants provided their written informed consent to participate in this study. Written informed consent was obtained from the individual(s) for the publication of any potentially identifiable images or data.

## Author contributions

DL: conceived the experiments. ZZ: designed the experiments and wrote the manuscript. JX and XC: provided guidance to the counterpart manuscript. All authors contributed to the article and approved the submitted version.
